# Assessing Continuity of Care for Postpartum Women in Standard and Home Visiting Service Delivery Models: Insights from a Lithuanian Study

**DOI:** 10.3390/healthcare14040477

**Published:** 2026-02-13

**Authors:** Ilona Tamutienė, Vaida Auglytė, Milda Naginevičiūtė, Rita Buitvydė, Aurelija Blaževičienė

**Affiliations:** 1 Department of Public Administration, Faculty of Political Science and Diplomacy, Vytautas Magnus University, 44248 Kaunas, Lithuania; ilona.tamutiene@vdu.lt (I.T.); vaida.auglyte@vdu.lt (V.A.); 2 Vytautas Kavolis Transdisciplinary Institute for Social Sciences and Humanities, Vytautas Magnus University, 44248 Kaunas, Lithuania; 3 Health Research Institute, Faculty of Public Health, Lithuanian University of Health Sciences, 47181 Kaunas, Lithuania; 4 Department of Nursing, Faculty of Nursing, Medical Academy, Lithuanian University of Health Sciences, 50106 Kaunas, Lithuania; milda.nagineviciute@lsmuni.lt; 5 Women and Men Equality Group, Ministry of Social Security and Labour, 03162 Vilnius, Lithuania

**Keywords:** postpartum, continuity of care, service delivery model

## Abstract

**Introduction**: A woman’s health and her child’s development are greatly affected by the responsiveness and support of the health system throughout the postpartum period. While various scholars have analysed the qualities of continuity of care and their effects during that phase, this article aims to reveal women’s experiences of postpartum care by analysing the impact of continuity of care through home visiting (HVCoC) versus standard care. **Methods**: Semi-structured interviews have been conducted in a qualitative study with 19 mothers of children under 1 year of age, who meet at least one criterion, such as living in poverty, being under 18 while giving birth, lacking permanent housing, residing in crisis centres due to domestic violence, or giving birth for the first time. All participants of the study have received either standard care or continuity of care through home visiting within the HVCoC model project. **Results**: The study has shown that women’s postpartum care experiences depend on the service delivery model. The standard care model, compared with the HVCoC model, has led to negative experiences for women across three dimensions: Relational, informational, and management continuity of care. **Conclusions**: While existing research has concluded that adequate postpartum support is related to the continuity of care model, this study’s findings reveal how different care organisation models affect the value women receive from their healthcare. Decision makers should develop postnatal care services that ensure continuity of care throughout pregnancy and the postpartum period by providing access to the same healthcare specialist for ongoing care.

## 1. Introduction

The postpartum period is a critical phase in a woman’s life. Commonly, postpartum is defined as the period from the child’s delivery to 6 months [1]. Various physical, mental and social health challenges mark this period [2,3,4,5]. Poor maternal health during the postpartum period can negatively affect a child’s cognitive, social, and emotional development [6]. Therefore, ensuring adequate healthcare and support for women during this period is crucial for the well-being of both mother and child. In this context, the search for high-quality service delivery models of care is an essential contribution of researchers to the problem.

The Continuity of Care (CoC) model is receiving increasing attention to improve health outcomes for women and children during the postpartum period [7,8,9,10,11]. Within this framework, the CoC is conceptualised as the extent to which a series of distinct healthcare events is perceived as coherent, interconnected, and aligned with the patient’s medical needs and personal circumstances [12]. In the postpartum context, successful support is linked to the relational, informational and management continuity of care [13].

Within this framework, a study conducted by [9] in a midwifery clinic in Sweden has implemented the coordinated postnatal care model to improve support and continuity of care for new mothers. Specifically, this model focuses on providing expanded attention to midwifery care during the pregnancy and postpartum periods. Moreover, the model includes early postnatal checks over the phone and clinic visit options tailored to the mother’s priorities. The study has found that the coordinated postnatal care model improves continuity, accessibility, and empowerment for mothers, enabling them to increase satisfaction and confidence. Furthermore, a well-organised care model strengthens the woman-midwife relationship, which, in turn, promotes postnatal planning and fosters satisfaction and trust. Overall, implementing this CoC model has improved maternal health and spurred exploration of alternative care options in Sweden, underscoring its positive impact [9]. Similarly, other researchers, for instance D’haenens et al. (2020), have highlighted the positive effect of the CoC, which includes breastfeeding, maternal and neonatal health, and the mother’s mental well-being [10] explicitly. Across both studies, the findings show that women place great value on the CoC, especially in terms of the midwife’s role. In contrast, women who have experienced fragmented care models identify numerous limitations and a lack of continuity [14]. Despite the positive outcomes of the CoC, there are several barriers to its implementation, especially structural ones [15]. Therefore, a strong theoretical and empirical foundation is needed for robust and stable engagement in the CoC. Given that the CoC is a women-centred model of maternity care, the health policy context and the transfer of knowledge about the CoC model to policy-makers, managers, and frontline practitioners are critical for its implementation [7]. The development of the CoC requires a comprehensive understanding of its importance, the provision of resources and a favourable environment [16].

It is vital that home visit CoC programmes are given due consideration. Not only do they have positive outcomes for women and their babies [17], but they also benefit the health system as a whole: Women receiving home visit services are less likely to have used emergency medical services for their infants [18]. Examining women’s perspectives in this context is particularly important, as it sheds light on the role of health services in helping women to cope with a range of health problems affecting both themselves and their babies.

Building on the above, this article aims to reveal women’s experiences of postpartum care by analysing the impact of continuity of care through home visiting (HVCoC) versus standard care. We examine how different care organisation models affect the value women receive from their healthcare. The study has been conducted in Lithuania and is of great relevance both nationally and for countries that have not yet developed the HVCoC model in primary healthcare during pregnancy and the postpartum period. This context provides an opportunity to examine the added value of the HVCoC. Before introducing the methods in the following section, we will briefly present the general context of healthcare and social support for pregnant and postpartum women in Lithuania, highlighting the standard care and HVCoC service delivery models.

### General Context of the Healthcare and Social Support for Pregnant and Postpartum Women with Children in Lithuania

Lithuania is a Baltic country that regained its independence from Soviet occupation in 1990. It is also a member of the European Union and NATO since 2004. At the beginning of 2024, Lithuania had a population of 2886 million inhabitants [19]. The GDP per capita was EUR 26,987 [20]. Lithuania has experienced a decline in fertility rates since 1990. The total number of births in Lithuania has been declining from 30,065 in 2015 to 18,673 in 2024 [21].

In the 7th month of pregnancy, women are eligible for maternity leave, which lasts approximately 70 days before the expected delivery and 56 days thereafter. In the case of a complicated delivery or the birth of more than one child, post-delivery leave can last for 70 days. During this period, women receive a maternity allowance amounting to 77.58% of their previous gross salary. However, if the mother has not accumulated 12 months of the qualifying work period in the last 24 months, she is only eligible for a lump-sum benefit, which in 2025 amounted to EUR 450.10 [22]. After the child is born, the father can take paternity leave, which lasts for 30 days. The father can use this option until the child turns one year old. During paternity leave, he receives the paternity allowance amounting to 77.58% of his gross salary [23]. The state provides a one-off cash benefit for every newborn child in Lithuania, which was EUR 770 in 2025, as well as a monthly payment, which amounted to EUR 122.5 in 2025. Families with low income, disabled children, or those raising multiple children receive an additional monthly allowance, which was EUR 72.1 in 2025 [24]. Either the child’s mother, father, or grandparents are entitled to childcare leave after the maternity/paternity leave. The childcare benefit payment depends on the chosen duration thereof. If childcare leave is taken until the child is 18 months old, the monthly childcare benefit will be 60% of gross salary; if the leave is taken until the child turns 24 months old, the benefit will amount to 45% of gross salary during the first 12 months, and 30% for the remaining 12 months. Childcare leave can be granted for up to 36 months, but the last 12 months (from 2 to 3 years old) will not be paid.

Pregnant women in Lithuania receive three levels of prenatal care. At the primary level, the responsibility for this care lies with a GP, an OB-GYN, or a midwife. If, during pregnancy, high-risk factors are present or develop, as evaluated at each consultation by a maternal health specialist, it becomes imperative to refer the pregnant woman to an OB-GYN for further evaluation. If the healthcare specialist identifies no risk factors during the ongoing pregnancy, there are planned preventive visits (recommended average number of visits is 7 for multiparous women, 10 for nulliparous women), where the medical history is collected, blood pressure and body mass index are measured, the required tests are performed, such as complete blood count, glucose tolerance, urine and urine culture tests, and the woman is referred to an OB-GYN for ultrasound or other specialised consultations. From the 24th week of pregnancy, the height of the uterine fundus is measured, and foetal heart rate is assessed. From the 36th week of pregnancy, a foetal position assessment is done. In case of any risk, visits to specialists are scheduled as indicated [24]. At 41 weeks of gestation, a decision is made either about scheduling the next visit or hospitalisation. In a typical case, new mothers are advised to have a gynaecological check-up 6 weeks after giving birth. This care procedure will be hereinafter referred to as the standard care model.

As maternal healthcare through home visits in Lithuania used to be limited to one postpartum visit, the Ministry of Health of the Republic of Lithuania initiated and implemented the Home Visiting Project (Development of a Model for Family Visits in the Provision of Early Intervention Services, No. EN03-2-SAM-TF-002). During the project implementation, home-visiting professionals (with a background in nursing or midwifery), who had completed specialised training, provided services to 325 families across 12 municipalities in Lithuania. Following the model developed by researchers at the Lithuanian University of Health Sciences, the visits included home-based support to pregnant and postpartum women on pregnancy and childbirth preparation, child and maternal healthcare, child immunisation, breastfeeding, child development, emotional attachment, a safe environment, and other topics. 13 healthcare service providers—Family Visiting Specialists (FVSs)—were required to visit families up to 64 times (14 home visits during the woman’s pregnancy, 28 visits from the baby’s birth to the age of 12 months, and 22 visits from month 13 until the child turns 2 years old). The FVSs were accessible 24/7 by phone or messenger. In this study, the project is referred to as the HVCoC model.

## 2. Research Framework and Method

**Figure 1:**
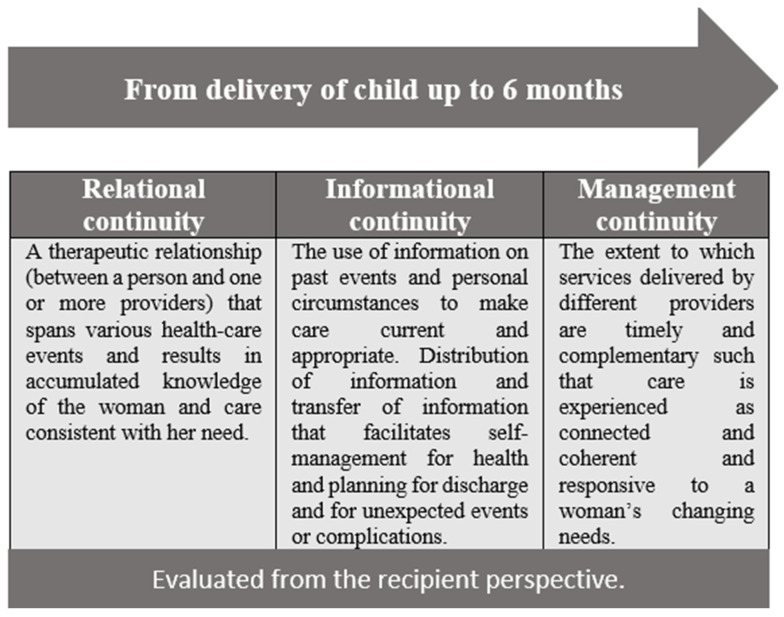
The CoC model for postpartum women.

The research design is based on a qualitative approach, which involves the CoC model developed by Haggerty et al. [12] and Eikemo et al. [9] (see Figure 1). It encompasses the individual patient’s experience of interconnected and coherent care over time and includes three key types of continuity: Relational, informational, and management continuity of care.

The essence of the relational continuity lies in the sustained connection between the healthcare or social care provider(s) and the woman, ensuring a sense of familiarity and trust throughout her care journey from pregnancy to postpartum.

The informational continuity involves using past information and individual context to customise current care. Sharing relevant information helps facilitate independent health management, support self-care, discharge plan and prepare for unexpected situations. This aspect ensures contextually relevant care, based on a comprehensive grasp of medical history and personal situation. Management continuity ensures that care is smoothly coordinated among providers, making it integrated, responsive, and aligned with the individual’s needs. Collectively, these three dimensions form a comprehensive CoC model that addresses the relational, informational, and management aspects contributing to a well-rounded, integrated healthcare experience for women.

### 2.1. Method of Semi-Structured Qualitative Interviews

A semi-structured interview method enables an in-depth exploration of participants’ experiences. This involves asking pre-prepared open-ended questions and follow-up questions during the interview. The semi-structured interview guidelines were developed collegially by the research team. The guide was designed to elicit women’s subjective experiences during pregnancy, childbirth, and the postpartum period. The identical key topic strands were applied to each chronological stage: Health and maternity issues experienced; support and services received; experience with provided services, including their responsiveness to women’s needs, service delivery process, timeliness, and perceived effectiveness; and significance thereof for women’s and children’s health and quality of life. This structure enabled a systematic comparison of experiences across time periods while maintaining the flexibility of the interviews and openness to participants’ stories.

Prior to the main data collection, two pilot interviews were conducted to assess the clarity, sequence, and suitability of the interview guide for the research objectives. Insights gained from the pilot interviews were discussed within the research team and used to refine the interview guide.

### 2.2. Participant Recruitment and Sampling

Given that this study aimed to compare the experiences of women who received standard care with those who participated in the Home Visiting Project, general criteria for participant selection were defined. These included mothers raising children under 1 year of age, who meet at least one criterion, such as living in poverty (receiving social benefits), being a minor (under 18 while giving birth, not having permanent housing or living in crisis centres due to domestic violence, or giving birth for the first time. The study groups were categorised according to the service delivery model received by participants: Standard care or HVCoC.

The study participants were selected using purposive sampling. Women with standard care experiences were reached through mediation by healthcare providers, including social workers, and by NGO specialists who assisted pregnant women and mothers with children meeting general selection criteria. The FVSs helped reach women who had experience with the HVCoC service. Once potential participants were contacted, they were given information about the study, and informed consent to participate in a semi-structured interview was obtained before the interview. Sampling, interviews, and data analysis were conducted in parallel and continued until data saturation, when additional interviews no longer provided new substantive experiences or topics. Achieving data saturation was the criterion for terminating further participant selection.

### 2.3. Data Collection Procedures

The interview process unfolded in three sequential stages. The first stage included an introduction and an ethical discussion. The participants were assured that their anonymity and confidentiality would be maintained, and that they could withdraw from the interview if they felt the need to do so. After obtaining participants’ consent, audio recording devices (specifically a phone app) and a voice recorder were used. In the second stage, the main interview questions were presented in a semi-structured manner, using open-ended and follow-up questions to elicit detailed experiences during pregnancy, childbirth, and the postpartum period. In the third, final stage, participants were invited to add any information they considered necessary that had not been previously discussed, after which the interview was respectfully concluded.

The interviews were conducted by the authors 1 and 2, both of whom had the required experience in conducting qualitative research. The study participants were recruited from six administrative units in Lithuania. The research was conducted from September 2022 to October 2023. The venues for the interviews were arranged based on the participants’ wishes: At the university, in crisis centres, and in neutral locations free of extraneous stimuli.

The conducted interviews lasted, on average, 1 h and 28 min. The shortest interview lasted 35 min, while the longest interview lasted 3 h and 53 min. The interviews were transcribed manually, and no additional coding software was used in this study.

### 2.4. Participants

The study involved 19 mothers with children under 1 year old. The average age of the mothers was 29, ranging from 16 to 42. Among them, 12 mothers were raising their firstborns, 5 were raising two children each, 1 was raising 5 children, and 1 was raising 3 children. 10 mothers participated in the Home Visiting Project and received HVCoC services both before and after giving birth. The remaining 9 mothers received standard care from the national healthcare system [See Table 1].

### 2.5. Data Analysis

Data analysis was conducted in stages, applying the principles of qualitative content analysis [25,26].

All the authors got access to the collected data. Two researchers then performed detailed independent coding, creating the conditions for collegial discussion and management of subjectivity. At first, a conventional qualitative content analysis was performed, in which two researchers independently conducted open coding, and codes and initial categories were inductively derived directly from the interview data. After each series of interviews, the newly derived codes and categories were discussed in collegial meetings with the research team to harmonise interpretations and ensure analytical consistency.

During the analysis, systematic differences in participants’ experiences by service model began to emerge. Given these empirical insights, it was decided to move to directed content analysis, based on the CoC theoretical model (Figure 1). Using directed content analysis, the data were then sequentially analysed according to the main theoretical dimensions of the CoC. To capture all possible occurrences of the phenomenon in question, text identified within each of the three categories was highlighted without coding. This approach was adopted because Hsieh & Shannon (2005) [25] noted that it might increase the study’s trustworthiness. No new categories emerged during the analysis, as the existing categories were deemed sufficient to capture the diverse experiences of the study participants across the two service models.

The results of the study were organised according to the theoretical dimensions of the CoC, enabling comparisons and interpretations of women’s experiences throughout different care contexts. The relevant quotes were selected during collegial discussion to reflect the participants’ typical and recurring experiences (see Appendix A).

### 2.6. Trustworthiness and Rigour

The trustworthiness of the study was ensured throughout the research process [27]. Peer debriefing was implemented to strengthen the study’s credibility, with interpretations emerging during data collection and analysis, and key analytical decisions regularly discussed within the research team. This facilitated critical examination of underlying assumptions and helped reduce the influence of individual interpretations.

Dependability was enhanced through independent coding, in which two authors coded the data independently and subsequently discussed and aligned their interpretations, as well as through analytical triangulation, which compared research participants’ experiences across different care models and chronological periods.

Confirmability was reinforced by applying reflexivity, with researchers continuously reflecting on their roles and the potential influence of their perspectives on the research process and data interpretation, and by grounding analytical conclusions in empirical data.

### 2.7. Research Ethics

The research protocols were approved by the Committee on Compliance with Professionalism and Ethics in Scientific Research of the Faculty of Political Science and Diplomacy of Vytautas Magnus University (No. VAK03052, 3 May 2022). Participation in the study was voluntary, and participants could withdraw at any time without negative consequences. All data collected was anonymised and processed in accordance with data protection requirements, ensuring the participants’ anonymity. Given the sensitive nature of the study, interviews were conducted in a manner that respected participants’ well-being and emotional safety.

## 3. Results

### 3.1. Relational Continuity of Postpartum Care

Relational continuity differed substantially between the two care models, particularly regarding emotional support, continuity of professional relationships, and perceived accessibility of care in the postpartum period. Women who had no contact with NGO support services and did not participate in the Home Visiting Project generally lacked relational continuity of care. Although they valued the physical care received during pregnancy, they consistently reported that their emotional needs were insufficiently addressed in the postpartum period. At the same time, these women expressed a preference for continuity with the same healthcare professional at the routine six-week postnatal visit, indicating an unmet need for the ongoing therapeutic relationship.

In contrast, women who received support from NGOs and FVSs reported positive relational continuity. Relationships established during prenatal care and the trust developed with professionals continued into the postpartum period, increasing women’s confidence and willingness to seek help after discharge from the maternity hospital. The participants highlighted the importance of sustained emotional support, individualised attention to their health, and practical guidance related to infant feeding and childcare. These contrasting experiences illustrate how different care models shape the availability and quality of relational support in the postpartum period (see Table 2).

Trustful relationships built during pregnancy also facilitated greater perceived accessibility of professionals beyond formal working hours. The participants emphasised that establishing trust took time and could not be achieved in a single visit. Timely and flexible access to the FVSs was therefore viewed as a key element of effective relational continuity. Overall, relational continuity remained fragmented and episodic in the standard care model, whereas the HVCoC model enabled sustained, trust-based relationships that enhanced women’s emotional security and access to timely support.

### 3.2. Informational Continuity

Informational continuity differed significantly between the two care models, particularly regarding access to timely professional guidance, clarity of care pathways, and perceived reliability of information in the postpartum period. Women generally reported receiving standard care during pregnancy; however, significant informational gaps emerged after discharge from the maternity hospital. Despite prenatal training, many participants felt underprepared for real-life postpartum situations and uncertain about when to seek help for common health concerns and where to get it. In the absence of timely professional advice, online resources became an essential alternative source of information.

The participants’ accounts indicated that trusting relationships established during pregnancy played a central role in maintaining informational continuity. Women who participated in the pilot Home Visiting Project consistently valued the information provided by the FVSs regarding both their own health and their children’s care. In contrast, women who did not participate in the project frequently reported uncertainty about where to seek support when difficulties arose (see Table 3).

Women who did not receive the FVSs’ services nevertheless valued the informational leaflets distributed in some municipalities, which provided basic guidance for early childcare. In the absence of structured professional support, the study participants often sought information independently when problems arose. Some women reported that contact with NGOs facilitated the informational continuity after childbirth; however, they emphasised that awareness of such support options should be established before giving birth to enable timely access to assistance. Overall, the informational continuity remained fragmented in the standard care model, with women relying primarily on self-directed information seeking, whereas the HVCoC model facilitated timely, personalised, and trusted access to professional guidance.

### 3.3. Management Continuity

Management continuity differed substantially between the two care models, particularly in service coordination, follow-up care accessibility, and the continuity of professional responsibility in the postpartum period. The study participants described significant structural gaps in postpartum care, including limited publicly funded follow-up visits and unclear responsibility-sharing among primary care providers. In practice, a single postnatal visit six weeks after childbirth was perceived as insufficient, often resulting in fragmented care pathways, delayed support, and reliance on emergency services, particularly for vulnerable women lacking informal support. Women with sufficient financial resources reported seeking private services, whereas such options were inaccessible primarily to socially vulnerable mothers.

Women who interacted with social workers and child protection services often perceived these visits as controlling rather than supportive, whereas the FVSs’ support was experienced as needs-based and relational. Some participants nevertheless highlighted the importance of coordinated intersectoral collaboration in complex situations, such as cases involving domestic violence, where timely referral pathways and clear professional responsibilities were critical for adequate support (see Table 4). Although separate sectors delivered services, the participants emphasised that continuity of professional relationships and clarity about available support enabled timely help-seeking and enhanced the management continuity.

Women who received the FVSs’ services valued the opportunity to receive coordinated care for themselves and their children within the home setting, reducing the need for multiple institutional visits and unnecessary referrals to GPs or emergency care units. In complex risk situations, mothers reported contact with various agencies; however, due to their specialised expertise in maternal and child health, the FVSs were consistently perceived as the most relevant and supportive professionals. Some participants nevertheless experienced involvement of social services as burdensome or controlling, underscoring the need for improved intersectoral coordination and person-centred management continuity.

Overall, management continuity remained fragmented in the standard care model, characterised by limited follow-up, unclear professional responsibility, and weak intersectoral coordination, whereas the HVCoC model supported more integrated, accessible, and responsive management continuity of postpartum care.

## 4. Discussion

We have examined how different care organisation models affect the value received by women from their healthcare providers. Our qualitative research results show that women’s experiences depend directly on the service delivery model applied. The standard care model, which does not include continuous home-based services and is limited to one specialist visit in the postpartum period, does not provide opportunities for women with infants to receive timely assistance and avoid preventable problems they and their infants encounter. The experiences of the study participants who had received services under the HVCoC model have shown the advantages of timely, continuous and needs-based care compared to the standard care model. The results of our study are very consistent with those of Eikemo et al. [9] and Schwind et al. [27]. The HVCoC has triggered changes, and ways are being explored at the national level to fund and make these services broadly accessible. The Ministry of Health of the Republic of Lithuania has scaled up the project services within the national healthcare system. The introduction of an FVS to the GP’s team has served as a management strategy to ensure the continued sustainability of the service [28].

Relational continuity

Relational continuity in the therapeutic relationship between care providers and women under research depended not only on personal factors such as trust and professionalism, but also on the structural ones. In the standard care model, the study participants valued physical healthcare services and were satisfied with them, but reported neglect of emotional healthcare, which prevented the formation of a trusting, sincere relationship between the patient and the specialist. On the contrary, the pre- and postnatal service scheme under the HVCoC model facilitated continuity of the relationship between the FVSs and women. The experiences of mothers who received the HVCoC services revealed that establishing a trusting relationship with the FVS during pregnancy was key to the relational continuity of care in the postpartum period. The importance of trust and relational continuity in the overall evaluation of the CoC model services is also emphasised by Perriman et al. (2024) in the systematic review and meta-synthesis [29]. Our research has revealed that relational continuity also helps informational continuity, because trust-based relationships enable women to seek information and help when the need arises. Taken together, the findings suggest that the added value of the HVCoC model lies not only in increased service availability, but in its organisational design, which enables stable care provider–woman relationships, timely access to care, and responsiveness to emerging needs across the postpartum period. These organisational features, by reinforcing relational, informational, and management continuity, appear to underpin women’s perception of the HVCoC model as particularly supportive.

Informational continuity

The analysis of women’s experiences receiving standard care services has shown that providing information and education only during pregnancy is insufficient. The postpartum period is a challenging time, as additional questions arise in real-life situations. This aspect of women’s experiences highlights the importance of the FVSs’ informational continuity when women face real-life challenges. The mothers who trusted the FVS and knew that they could turn to them for information and support were very appreciative of this opportunity. Our study is related to the study by Nazarenko et al. [30], which is based on a literature review. The authors of that study found that, in the USA, women reported feeling unprepared for numerous postpartum experiences, including depression, anxiety, physical recovery, breastfeeding, and infant care, and also noted the need for earlier postpartum visits and improved outpatient support.

Our study found that sharing relevant information has increased women’s self-confidence and empowered them to take care of their own health and that of their babies. Additionally, information and counselling about maternal and infant health, and the perceived security during postpartum visits, reduced maternal stress and the need for visits to the emergency room and GPs. We cannot determine the extent of this from the qualitative study. However, Sorbara et al. [29] report that a combination of 24/7 postpartum care and home visits is associated with a 22% lower risk of emergency department visits, compared with traditional clinic-based care.

Management continuity

Management continuity ensures that care is well coordinated among various providers, is integrated, responsive, and relevant to individual needs. The experiences of women who did not participate in the HVCoC model showed that they were left on their own when they returned from the maternity hospital after obstetric care. Women’s experiences suggest that a single home visit, which is child-focused, is not sufficient to ensure the postpartum CoC. The COVID-19 quarantine might have influenced this model. However, currently, it reveals a structural problem, since one visit is not enough.

For both social and health problems, family interventions must provide coordinated support. However, the analysis of informational continuity revealed that women lacked information about NGOs providing support to pregnant women and mothers with babies. This situation shows that, in responding to the needs of women and their babies, there is still a lack of coordination and integrity among service providers.

Women who received services through the HVCoC model and social and child protection interventions indicated that not all support was tailored to their needs and the needs of the child. When they encountered interventions from social workers and child protection services, they experienced them as controlling rather than supportive. The interviewed women revealed a clear need for support from the FVSs in the postnatal period, including emotional support, breastfeeding support, practical childcare support, and support for their own health, rather than just an assessment of whether all this was being provided.

From a policy perspective, the findings indicate that implementing HVCoC at the primary care level requires sustained human resources, interprofessional coordination, and clear role allocation, particularly for FVS. Although the HVCoC model shows clear benefits, its wider implementation may be constrained by workforce capacity and the need for stable financing mechanisms. Conducted within the Lithuanian healthcare system, where HVCoC has only recently been integrated into routine postpartum primary care, this study’s findings may have limited direct transferability to other health systems. Nevertheless, the mechanisms identified—such as relational continuity, timely information exchange, and coordinated management—are likely relevant across different healthcare contexts and may help explain why women perceive HVCoC as more supportive in practice.

The study has shown that the most rigorous care and emotional support are needed in the first few weeks after childbirth. This need, which requires specialised knowledge in obstetrics and paediatrics, is met by the FVSs. Thus, among the different service providers, the FVSs’ contributions have proven most relevant to the needs of women and children at this stage. Barimani & Vikström point out that management continuity after childbirth is the most critical factor for ensuring the CoC [13]. However, we have identified relational continuity with the FVSs as a crucial factor in ensuring the CoC and coordinating community care during the postpartum period. This is consistent with the study by Nazarenko et al. (2024) [30].

A key strength of this study lies in its focus on the perspectives of women who received services, allowing a comparative analysis of two care models and revealing how different service designs respond to the needs of pregnant and breastfeeding women. From the participants’ perspective, the effectiveness of the HVCoC model stems from its design and management, which prioritise relational, informational, and management continuity. Early and consistent contact with the same healthcare specialist helped establish a clear and reliable care trajectory. These findings highlight the importance of continuity as a core principle of service delivery, dependent on primary care management and sufficient human and organisational resources. Furthermore, the study illustrates good practice, showing which care model best meets women’s needs and providing insights for policymakers and service managers on the conditions required for effective and sustainable implementation.

## 5. Limitations

There are several limitations to the study. First, it is based on a relatively small sample of the qualitative research. Second, the analytical findings presented in the discussions cannot be broadly generalised. Third, the COVID-19 management strategies might have influenced the results. This is particularly true for the women who took part in the study as representatives of the standard care model, as they were interviewed right after the end of the quarantine.

## 6. Conclusions

The service users’ experiences with the HVCoC model have highlighted its advantages in terms of continuity of care and in meeting the needs of mothers and children, compared with the standard care model. Of particular note is the relational continuity, as well as the availability of midwifery and paediatric expertise among service providers, which enables them to meet the needs of both mothers and children. Health sector decision-makers should design postnatal care services to ensure the CoC throughout pregnancy and the postpartum period. This will facilitate relational and informational continuity by providing access to the same healthcare specialist, empowering them to be the key player in coordinated community care during a period so vulnerable to maternal and infant health and development.

## Figures and Tables

**Table 1:** Demographic data of the research participants.

Participant Code	Gender	Age	Number of Children Raised	Place of Residence	Model of Care
M1	Female	28	1	City	Standard
M2	Female	23	1	City	Standard
M3	Female	42	5	City	Standard
M4	Female	29	2	City	Standard
M5	Female	29	1	City	Standard
M6	Female	32	2	City	Standard
M7	Female	16	1	Rural area	Standard
M8	Female	17	1	City	Standard
M9	Female	29	2	City	Standard
M10	Female	35	1	City	HVCoC
M11	Female	23	2	City	HVCoC
M12	Female	25	1	City	HVCoC
M13	Female	37	1	City	HVCoC
M14	Female	29	1	Rural area	HVCoC
M15	Female	39	1	City	HVCoC
M16	Female	21	3	City	HVCoC
M17	Female	27	2	Rural area	HVCoC
M18	Female	32	1	City	HVCoC
M19	Female	32	1	City	HVCoC

**Table 2:** Contextualising the experiences of women with and without relational continuity of care.

Experience Without Relational Continuity (Standard Care Model)	Experience with Relational Continuity (CoC Model)
“In fact, I read extensively. Furthermore, I had a keen interest; I watched numerous videos and consulted many friends to determine the best approach. When I faced a problem, it had a major impact on me. There was also a situation where I experienced difficulties with breastfeeding. My breasts were filled with milk, but the baby couldn’t eat much yet, so they became very hard. Furthermore, it was very painful. To be more precise, I felt excruciating pain. When I faced this issue, I couldn’t stop crying. I immediately started making phone calls. I was calling everyone for advice because I was lost. It was evening, the baby was crying, and I was under a lot of stress. So, I got a suggestion to use cabbage leaves. That was the moment when I started using more traditional methods.” (M2) “No one is interested in the emotional state of a woman after childbirth. Everyone is focused on the newborn and its health. But it’s very important that someone takes care of the mother so that she can take care of the baby.” (M5)	“At the beginning of breastfeeding, she was incredibly helpful. She always guided me with care, patience, and attention to make breastfeeding as comfortable and as pain-free as possible. Step by step, she provided clear verbal instructions, showed me how to breastfeed properly, and pointed out mistakes to avoid. She explained in detail how to breastfeed and latch correctly, and showed proper baby-holding positions to prevent nipple pain and other discomfort. In case of emergencies, important questions, or issues related to the baby, the specialist would answer questions and help resolve problems, regardless of the time or day of the week. She always responded to messages or calls.” (M10)

**Table 3:** Contextualising the experiences of women with and without informational continuity of care.

Experience Without Informational Continuity	Experience with Informational Continuity
“And, you know, it would have been easier if mothers knew where to turn to after giving birth because there’s definitely a lack of information about where you can go. Where to seek help if there are problems with breastfeeding or other problems, whether financial or social? So, yeah, I think there’s a lack of this kind of information…” (M3) “There is really a lack of information, and there is just a very high occupancy rate of specialists, of GPs, and it is just challenging to get the information you need, because of things that are not fully understood. Because the GP has very little time. There is no, just not enough information, not enough time for a consultation and just, well, it’s just not possible to find out everything that I want. There isn’t enough time for specialists. Maybe there should be a 30 min consultation for young children instead of 15. Maybe there should be a home visit from the GP. So maybe these things were missing, that there was just no home visit, and there was just nowhere to go for breastfeeding advice, because I had breastfeeding problems too, and I didn’t know where to go for breastfeeding advice. Just all these things.” (M1)	“When our first child was born, I had quite a few questions about the baby, its emotional state, feeding, and ultimately, postpartum well-being. Interactions with the family visiting specialist greatly relieved my postpartum experience. Discussing the child’s development and any concerns with the specialist, Simona B., was very helpful. I liked the fact that I could simply send a message via Facebook or Messenger and receive a prompt response. She is undoubtedly competent and can address any questions that may arise. For instance, recently, when my baby had a rash, I sent a photo and received advice on what it might be and how to treat it.” (M11) “I received support whenever questions arose. I could always reach out and ask whether I was doing everything right, at any time, including weekends.” (M13) “For all my pressing questions, I receive additional information from the FVS and guidance on where to find it.” (M16)

**Table 4:** Contextualising the experiences of women with and without management continuity of care.

Without Management Continuity	With Management Continuity
“…he has beaten me. And then I went to the clinic. Well, to my X clinic. All woozy, all scared. And I asked that, well, maybe I could see my GP, so that she could, well, let’s say, record those, well, those health problems, yeah. And then the receptionist said to me: ‘You know, you have to go to the X place, there’s a special centre there where they’ll admit you, they’ll examine you, and that forensic doctor will record it’. But I, at that time, I was in such a state that I did not want to go anywhere, nor look for where that X place was. And I… I left. And I wasn’t directed to any external services. I think there’s some kind of psychologist at the X clinic. Because there’s a mental health centre down there too. I wasn’t stopped, and I wasn’t taken to any specialist. I came, I asked, they said no, and I went on wandering into the street. They should then have instructions on what to do with it. Because in this case, it seems to me, it is irrelevant whether I was pregnant or whether I was with a baby doll. If a person comes in like this, lost, you can see that the whole thing is practically inadequate. They should have instructions on what to do with that person, how to help them, how to keep them, how to direct them, you know, where they need to go. Or maybe my state of mind is such that I’m going to jump off the bridge when I’m driving to the X place? Just like that, right?” (M3)	“I experienced significantly less stress as the specialist provided reliable and professional information. I didn’t need to bother my doctor or go to the emergency care unit. In the final months before giving birth, I was overwhelmed by prenatal depression, but it rapidly responded to the guidance I received, and everything improved. I got a lot of information about which medications and creams to use. When it came to breastfeeding, I received comprehensive guidance on what to do and how to do it best. My specialist (authors’ remark: the FVS) helped me avoid visits to my family doctor and the emergency room.” (M19) “I don’t need the ‘help’ of social workers and child protection services, my FVS is enough for me.” (M17)

## Data Availability

The original contributions presented in this study are included in the article. Further inquiries can be directed to the corresponding author.

## Appendix A. Main Categories and Sub-Categories of Postpartum Care Continuity

**Relational continuity**
Women who had no connection with the assistance provided by NGOs and did not participate in the HVCoC during pregnancy did not experience any relational continuity.
Pregnant women assisted by NGOs and by the FVSd experienced and acknowledged trusted relational continuity during the postpartum period.
The relationships and trust established during pregnancy care persisted in the postpartum period and facilitated informational continuity, which, in turn, affected management continuity.
**Informational continuity**
Despite planned educational sessions during pregnancy, women returning from maternity hospitals felt insufficiently informed, were self-conscious, and lacked maternity skills when encountering real-life situations.
In the standard care model, women were unsure when and where to seek help for breastfeeding problems, breast issues, bleeding, or other health concerns. In real life, they needed information and guidance. HVCoC met this need.
Women felt the need for information on support available from other institutions, including emotional and financial support from NGOs, as women were not always referred to such resources. It was not a case for women in HVCoC.
An information leaflet with basic guidance on whom to contact for specific issues, given to a new mother along with the essentials for the baby provided by the maternity hospitals, was considered a valuable resource.
The real-life information provided by the FVSs was positively experienced by the women who received home visits. They valued the fact that specialists were accessible when they needed information.
During the postpartum period, in the absence of direct, qualified information from specialists, online resources became a significant source of information.
A prior relational connection between the service provider and women enhanced informational continuity.
**Management continuity**
In the standard care model, timely services and need-based care were hindered by systemic gaps. Maternal healthcare received insufficient attention, as merely a single visit 6 weeks after childbirth proved inadequate.
Standard care sectors operated in isolation, often failing to align with where they were needed. In postpartum care, responsibility remained with either a GP or a midwife. However, practical scenarios arose in which neither of these professionals was involved.
For some women, home visits by social workers or child protection services during the postnatal period were experienced more as a form of control, while the FVSs’ care was seen as a real assistance that met their needs.
